# Study of quantum dots (CdS, ZnS) toxicity in *Danio rerio*: preliminary results

**DOI:** 10.1080/07853890.2021.1897422

**Published:** 2021-09-28

**Authors:** Beatriz Matos, Marta Martins, Mário S. Diniz

**Affiliations:** a UCIBIO, Department of Chemistry, Faculdade de Ciências e Tecnologia, Universidade NOVA de Lisboa, Caparica, Portugal; b MARE – Marine and Environmental Sciences Centre, Faculdade de Ciências da Universidade de Lisboa, Campo Grande, Lisboa, Portugal

## Abstract

**Introduction:**

Quantum Dots (QDs) are nanoparticles with potential applications in many industrial and biomedical areas (e.g. fluorescent dyes, LED). QDs have unique and remarkable properties, however, little is known about their toxicity [1]. Zebrafish (*Danio rerio*) has been the first vertebrate model in many research areas and is recognised as a good model in nanotoxicology [2]. This work aims to evaluate the toxicity of QDs (CdS and ZnS) in *D. rerio*, singly and combined, by determining a set of biomarkers (e.g. antioxidant enzymes).

**Materials and methods:**

Adult zebrafish (*n* = 50 (× 2); 0.3 ± 0.1 g weight; 2.7 ± 0.4 cm) were distributed by 4 glass containers (1 L) and exposed to different concentrations of QDs (0 ppb, 10 ppb, 100 ppb and 1000 ppb), for 7 days. Trials were performed in duplicate using 3 different groups exposed to: (1) ZnS-QDs, (2) CdS-QDs), (3) (ZnS-QDs + CdS-QDs). Oxidative stress was assessed by measuring catalase (CAT), glutathione-S-transferase (GST), lipid peroxidation (LPO), superoxide dismutase (SOD) and Total Antioxidant Capacity (TAC), as described previously [3,4]. Trials followed the 3Rs and animal welfare and were authorised by national authorities. Statistics was carried out using the Kruskal-Wallis test (STATISTICA 8.0, USA).

**Results and Discussion:**

Results show no significant differences between the 3 groups of experiments. Regarding enzyme activities (nmol/min/mg tot. protein), CAT the highest levels in fish exposed to 100 ppb Zn-QDs (45 ± 2) and the lowest levels were measured in controls (26 ± 4). GST showed the highest GST levels in fish exposed to 10 ppbZn-QDs (50 ± 1) and lowest levels in fish exposed to 1000 ppbCd-QDS (27 ± 16). SOD results (as % inhibition/mg tot. protein) showed highest values in fish exposed to 10 ppb QDs combined (89 ± 3%) and lowest levels in fish exposed to 100 ppb of Zn-QDs (35 ± 9). CAT, GST and SOD showed higher levels in fish exposed to 10 ppb and 100 ppb QDs. LPO showed non-sgnificant diferences TAC showed a trend to decrease in all groups exposed to different concentrations of QDs.

**Conclusions:**

These results suggest that QDs can induce moderate or low oxidative stress. The higher results observed in fish exposed to 10 ppb and 100 ppb of QDs can be due to QDs aggregation occurring at higher QDs concentrations, which can also affect the bioavailability of toxic ions released. Therefore, in this preliminary study, the lower concentrations of QDs seem to be more hazardous to Zebrafish.
